# Predictors of adverse outcomes using a multidimensional nursing assessment in an Italian community hospital

**DOI:** 10.1371/journal.pone.0249630

**Published:** 2021-04-15

**Authors:** Beatrice Gasperini, Gilda Pelusi, Annamaria Frascati, Donatella Sarti, Franco Dolcini, Emma Espinosa, Emilia Prospero

**Affiliations:** 1 Section of Hygiene and Public Health, Università Politecnica delle Marche, Ancona, Italy; 2 Geriatrics, Azienda Ospedaliera Ospedali Riuniti Marche Nord, Fano (PU), Italy; 3 School of Nursing, Università Politecnica delle Marche, Ancona, Italy; 4 Intermediate Care-Community Hospital, Loreto (AN), Italy; 5 ASUR Marche, Ancona, Italy; King’s College London, UNITED KINGDOM

## Abstract

**Background:**

There is growing evidence about the role of nurses in patient outcomes in several healthcare settings. However, there is still a lack of evidence about the transitional care setting. We aimed to assess the association between patient characteristics identified in a multidimensional nursing assessment and outcomes of mortality and acute hospitalization during community hospital stay.

**Methods:**

A retrospective observational study was performed on patients consecutively admitted to a community hospital (CH) in Loreto (Ancona, Italy) between January 1^st^, 2018 and May 31st, 2019. The nursing assessment included sociodemographic characteristics, functional status, risk of falls (Conley Score) and pressure damage (Norton scale), nursing diagnoses, presence of pressure sores, feeding tubes, urinary catheters or vascular access devices and comorbidities. Two logistic regression models were developed to assess the association between patient characteristics identified in a multidimensional nursing assessment and outcomes of mortality and acute hospitalization during CH stay.

**Results:**

We analyzed data from 298 patients. The mean age was 83 ± 9.9 years; 60.4% (n = 180) were female. The overall mean length of stay was 42.8 ± 36 days (32 ± 32 days for patients who died and 33.9 ± 35 days for patients who had an acute hospitalization, respectively). An acute hospitalization was reported for 13.4% (n = 40) of patients and 21.8% (n = 65) died. An increased risk of death was related to female sex (OR 2.25, 95% CI 1.10–4.62), higher Conley Score (OR 1.19; 95% CI 1.03–1.37) and having a vascular access device (OR 3.64, 95% CI 1.82–7.27). A higher Norton score was associated with a decreased risk of death (OR 0.71, 95% CI 0.62–0.81). The risk for acute hospitalization was correlated with younger age (OR 0.94, 95% CI 0.91–0.97), having a vascular access device (OR 2.33, 95% CI 1.02–5.36), impaired walking (OR 2.50, 95% CI 1.03–6.06) and it is inversely correlated with a higher Conley score (OR 0.84, 95% CI 0.77–0.98).

**Conclusion:**

Using a multidimensional nursing assessment enables identification of risk of nearness of end of life and acute hospitalization to target care and treatment. The present study adds further knowledge on this topic and confirms the importance of nursing assessment to evaluate the risk of patients’ adverse outcome development.

## Introduction

Intermediate transitional care models provide positive outcomes for older adults, contributing in functionality and quality of life improvements and reducing hospital admissions [1]. A Community Hospital (CH) is an intermediate care solution that has been introduced to promote the transition from the hospital to the community. Care is typically provided under the management of general practitioners. CHs ensure a rapid discharge from the hospital to a protected environment, patient safety and a convenient length of stay to achieve clinical stabilization [2]. Literature evidence shows that admission into a CH after hospitalization improves quality of life and reduces the length of hospital stay, the rate of hospital readmissions and mortality after discharge [3, 4]. CHs also admit patients that are referred by the general practitioner, because they cannot be treated at home, but do not need the intensity of hospital care (e.g. exacerbation of chronic diseases, antibiotic therapy).

CHs provide nursing care for people requiring a high level of supervision, the administration of drugs or interventions that would not be suitable for a nursing home or a home care setting [2].

Nurses have a high degree of autonomy and control over patient care within CHs [5], hence, nursing assessments are a key element of patient’s care pathway [6].

Nursing sensitive outcomes (NSOs) have been the subject of considerable research [7, 8]. The American Nurses Association has defined NSO as a measure of nursing care on patient care and patient outcome [9, 10], even if they are also correlated with patient morbidity and the care environment [11]. NSOs can be classified in three different categories: patient-related outcomes, nursing-related outcomes and setting-related outcomes [8]. Mortality and acute hospitalization are considered patient-related and setting-related outcomes, respectively [8]. The aim of this study was to assess the association between patient characteristics identified in a multidimensional nursing assessment and outcomes of acute hospital admission and mortality during the CH admission.

## Methods

### Study design, data source and participants

This was a retrospective observational study of patients who were consecutively admitted to a CH with 19 beds in Loreto (Ancona, Italy) between January 1st, 2018 and May 31st, 2019. The Strengthening the Reporting of Observational Studies in Epidemiology (STROBE) Statement guidelines for reporting observational studies were followed for the conduction of this study [12] (S1 Checklist).

Patients were managed by four general practitioners and three specialist physicians, 16 nurses and 16 healthcare workers. The nurse/bed and physician/bed ratio were 0.55 and 0.34, respectively, indicating that patients’ care was mainly provided by nurses. Nursing care was provided 24 hours for seven day a week. In Italy, the number of beds of intermediate care (including rehabilitation) was established at the Regional level. In the Marche Region the number is 0.74/1000 persons (Regional Law 735/2013). Nursing records of all patients were analyzed to assess factors associated with mortality and acute hospitalizations during CH stay.

### Main outcomes and covariates

Outcomes were mortality [11] and acute hospitalization (emergency department visit or in-hospital) [11] during CH stay. Each patient admitted into this CH underwent the standardized nursing assessment which included the following:

1. Sociodemographic characteristics (age, sex).
2. Nursing diagnosis defined using the North American Nursing Diagnosis Association International taxonomy [13].
3. Conley scale: a score≥2 indicates an increased risk of falling [14].
4. Norton score: a higher score indicates a lower risk of pressure sores [15].
5. Presence of a vascular access device. To our purpose we considered i) midline catheter (a peripheral line between 7.5cm–20cm in length), ii) peripherally inserted central catheter (PICC); iii) a short-term central vascular access device (CVC); iv) skin-tunneled catheter; v) an implanted port [16, 17]. Peripheral cannula (less than 7.5 cm in length) was not included in our analysis.
6. Presence of urinary catheter.
7. Presence of feeding tubes: nasogastric or percutaneous endoscopic gastrostomy (PEG) tube.
8. Presence of a stoma (colostomy or ileostomy).
9. Parenteral nutrition.
10. Presence of pressure sores.
11. Functional status (Activities of Daily Living, ADL scale) [18]: ability in moving, dressing, bathing, feeding, walking and urinary and fecal continence.
12. Comorbidities recorded from a list of clinical conditions coded in 7 categories: infective, hematological, neoplastic, orthopedic, pulmonary, cardiovascular, neuropsychiatric (dementia, depression, bipolar disorders, psychosis and other psychiatric conditions).

### Data analysis

Continuous variables were described as mean and standard deviation, and categorical variables were presented as frequency and percentage.

A descriptive analysis was performed to assess characteristics of patients according to 2 outcomes: acute hospitalization and mortality during CH stay.

A t-test was used to compare continuous variables. The chi-squared test was used to compare frequencies, using Fisher’s exact test as adjustment for expected frequencies less than 5.

A univariate analysis was performed to establish the association between mortality or acute hospitalization during CH stay and patient characteristics: age, sex, comorbidities, risk of falls (Conley score), risk of pressure sores (Norton scores), presence of urinary catheter, presence of any vascular access devices, feeding tubes (nasogastric or PEG), stoma (colostomy or ileostomy), parenteral nutrition, functional status (preserved ADL), presence of pressure sores at admission, nursing diagnosis defined in accordance with NANDA taxonomy (version 2009) assigned at admission.

Two binary logistic regression models were ultimately developed, using mortality and acute hospitalization recorded during CH stay as dependent variables. Regression models included variables that showed an association with the outcomes in the univariate analysis. Variables with a p value lower than 0.2 were included for conservative purpose. Variables with less than five observations were excluded. A stepwise technique was used. Statistical significance was considered for p< 0.05. Data analysis was performed with SPSS version 25 (Illinois, SPSS Inc. Chicago, IL, USA).

### Ethical statement

Given this was a retrospective observational study, ethical Committee of Marche Region does not require a formal approval. The study was performed in accordance with the Code of Ethics of the World Medical Association for experiments involving humans (Declaration of Helsinki) and research on health databases (Declaration of Taipei).

Patients and caregivers gave their written consent to use their personal data at their admission to the CH. Patient anonymity was respected during the process of data analysis and results reporting. The patients’ medical records were accessed anonymously, and a sequential number was used to identify each patient. A.F and F.D. are members of the Community Hospital staff.

## Results

A total of 298 patients’ records were analyzed. The mean sample age was 83 ±9.9 years (range 44–102), 60.4% (n = 180) were women. Pressure sores were identified on admission in 35.9% (n = 107) of patients and 65.4% (n = 195) of patients were at high risk to develop them. Some (84.2%, n = 251) patients were at risk for falls, 13.1% (n = 39) were under an enteral or parenteral nutrition and 41.6% (n = 124) had a vascular access device. The main comorbidities were cardiovascular (86.9%, n = 259) and neuropsychiatric (62.7%, n = 187). The main neuropsychiatric comorbidities were dementia and depression. The most frequent nursing diagnosis was risk of infection (i.e. urine infection, skin infection) (68.5%, n = 204), risk of falling (65.8%, n = 196), self-care deficit in bathing (62.4%, n = 186), impaired walking (60.1%, n = 179) and constipation (54%, n = 161) (Table 1, S1 Appendix).

**Table 1:** Patients’ characteristics gathered using a multidimensional nursing assessment.

	Total sample N = 298
Sex (F), n (%)	180 (60.4)
Age (years, mean±SD)	83±9.9
Length of stay (days, mean±SD)	42.8±36
Urinary catheter, n (%)	193 (64.8)
Vascular access device, n (%)	124 (41.6)
Pressure sores at admission, n (%)	107 (35.9)
Enteral nutrition, n (%)	28 (9.4)
Stoma, n (%)	12 (4.0)
Parenteral nutrition, n (%)	11 (3.7)
**Norton score (mean±SD)**	10.3±4
High risk, n (%)	195 (65.4)
Low risk, n (%)	58 (19.5)
Intermediate risk, n (%)	45 (15.1)
**Conley score (mean±SD)**	4.7±2
Conley> = 2 (high risk of falling), n (%)	251 (84.2)
**Activities of Daily Living preserved**, n (%)	
5–6	16 (5.4)
0–1	66 (22.1)
**Comorbidities**, n (%)	
Cardiovascular	259 (86.9)
Neuropsychiatric	187 (62.7)
Pulmonary	133 (44.6)
Orthopedic	99 (33.2)
Infective	68 (22.8)
Hematological	43 (14.4)
Neoplastic	33 (11.1)
Nursing diagnoses*, n (%)	
Risk for infection	204 (68.5)
Risk of falling	196 (65.8)
Deficit in bathing self-care	186 (62.4)
Impaired walking	179 (60.1)
Constipation	161 (54.0)
Risk for impaired skin integrity	152 (51.0)
Impaired transfer ability	149 (50.0)
Insomnia	121 (40.9)
Imbalanced Nutrition Less Than Body Requirements	119 (39.9)
Risk for unstable blood glucose level	109 (36.6)

*≥ 10 patient records.

At least 1 acute hospitalization was reported for 13.4% (n = 40) of patients and 21.8% (n = 65) died during CH stay. Variables associated with death (Table 2, S2 Appendix) were female sex (p = 0.031), older age (p = 0.005), length of stay (0.007), presence of pressure sores (p < 0.001), having a vascular access device (p < 0.001), urinary catheter (p = 0.011), parenteral (p = 0.003) and enteral (p = 0.003) nutrition, higher Conley score (p < 0.001) and lower Norton score (p < 0.001). Neuropsychiatric comorbidity was associated with mortality (p = 0.008). Nursing diagnoses associated with death were risk for infection (p<0.001), total urinary incontinence (p = 0.009), risk for impaired skin integrity (p = 0.013), difficulty in swallowing (p<0.001), risk of ab-ingestis pneumonia (p < 0.001).

**Table 2:** Patients’ characteristics in accordance with outcomes (mortality and acute hospitalization during CH stay).

	Mortality			Acute hospitalization		
	Survivors N = 233	Not survivors N = 65	P	No acute hospitalization N = 258	Acute hospitalization N = 40	P
Sex (Female), n (%)	134 (57.5)	47 (72.3)	0.031	158 (60.8)	23 (42.5)	0.652
Age (years, mean±SD)	81±10.2	85+9.7	0.005	83.2±9.8	76.4±11	<0.001
Length of stay (days, mean±SD)	46±36	32±32	0.007	44.5±35	33.9±35	0.880
Pressure sores, n (%)	71 (30.5)	36 (55.4)	<0.001	89 (34.5)	18 (45.0)	0.198
Vascular access device, n (%)	80 (34.3)	44 (67.7)	<0.001	100 (38.7)	24 (60.0)	0.011
Stoma, n (%)	11 (4.7)	2 (3.1)	0.741	8 (2.7)	5 (12.5)	0.019
Parenteral nutrition, n (%)	4 (1.7)	7 (10.8)	0.003	10 (3.9)	1 (2.5)	0.999
Enteral nutrition, n (%)	15 (6.4)	13 (20)	0.001	25 (9.7)	3 (7.5)	0.999
Urinary cathete, n (%)	143 (61.4)	51 (78.5)	0.011	165 (63.6)	29 (72.5)	0.291
Norton score (mean±SD)	11.3±3.6	7.25±3	<0.001	10.3±4	10.9±3	0.397
**Norton risk group**, n (%)			<0.001			0.899
Low risk	57 (24.5)	1 (1.5)		50 (19.4)	8 (20.0)	
Intermediate risk	45 (19.3)	5 (7.7)		40 (15.5)	5 (12.5)	
High risk	136 (58.4)	59 (90.8)		168 (65.1)	27 (67.5)	
**Conley score (mean±SD)**	4.4±2.6	6.1±2.5	<0.001	4.9±2.6	3.4±2.6	0.001
Conley> = 2, n (%)	187 (80.3)	63 (96.9)	0.001	225 (87.2)	25 (62.5)	<0.001
**ADL preserved <5**, n (%)	218 (93.6)	63 (96.9)	0.536	244 (94.6)	37 (92.5)	0.461
**Comorbidities**, n (%)						
Infective	52 (21.9)	17 (26.1)	0.517	56 (21.3)	13 (32.5)	0.132
Hematological	34 (14.6)	9 (13.8)	0.880	35 (13.6)	8 (20.0)	0.281
Neoplastic	24 (9.9)	10 (15.4)	0.254	30 (11.2)	4 (10.0)	0.999
Orthopedic	81 (34.8)	18 (27.7)	0.284	83 (32.2)	16 (40.0)	0.328
Respiratory	105 (45.1)	28 (43.1)	0.776	112 (43.4)	21 (52.5)	0.282
Cardiological	203 (86.7)	57 (87.7)	0.903	226 (87.2)	34 (85.0)	0.647
Neuropsychiatric	137 (58.8)	50 (76.9)	0.008	164 (63.6)	23 (57.5)	0.460
**Nursing diagnoses** *						
Risk for infection	146 (62.7)	56 (86.1)	<0.001	171 (66.3)	31 (77.5)	0.158
Constipation	124 (53.2)	37 (56.9)	0.596	150 (58.1)	11 (27.5)	<0.001
Total urinary incontinence	46 (19.7)	23 (35.4)	0.009	61 (23.6)	8 (20.0)	0.611
Risk for impaired skin integrity	110 (47.2)	42 (64.6)	0.013	136 (52.7)	16 (40.0)	0.134
Impaired walking	144 (61.8)	35 (53.8)	0.247	150 (58.1)	29 (72.5)	0.084
Difficulty in swallowing	45 (19.3)	27 (41.5)	<0.001	63 (24.4)	9 (22.5)	0.792
Self-care deficit in toileting	43 (18.4)	6 (9.2)	0.074	35 (13.7)	14 (35.0)	0.001
Risk for ab-ingestis pneumonia	25 (10.7)	21 (32.3)	<0.001	38 (14.7)	7 (17.5)	0.600
Risk for falls	155 (66.5)	42 (64.6)	0.774	175 (67.8)	22 (55.0)	0.111

*. Diagnosis reported in ≥ 10 patient records among patients who died or who had an acute hospitalization were presented. A full list of nursing diagnoses and their frequency is presented in Appendix 2.

TPN = total parenteral nutrition; NGT = nasogastric tube.

Younger age (p < 0.001), having a vascular access device (p = 0.011), stoma (p = 0.019), higher risk of falling (p = 0.001), constipation (p <0.001) and self-care deficit in toileting (p = 0.001) were associated with acute hospitalization.

Regression analysis found an increased risk of death correlated to female sex (OR 2.25, 95% CI 1.10–4.62), higher Conley score (OR 1.19; 95% CI 1.03–1.37) and having a vascular access device (OR 3.64, 95% CI 1.82–7.27). A higher Norton score was associated with a decreased risk of death (OR 0.71, 95% CI 0.62–0.81) (Table 3).

**Table 3:** Logistic regression model to assess factors associated with mortality during CH stay.

	P	OR	Confidence interval (95%)	
Sex (female)	0.026	2.25	1.10	4.62
Norton score	<0.001	0.71	0.62	0.81
Conley score	0.016	1.19	1.03	1.37
Vascular access device	<0.001	3.64	1.82	7.27

Model adjusted for: pressure sores at admission, feeding tubes, urinary catheter, psychiatric comorbidity; nursing diagnoses: ‘‘ risk for infection ‘‘ total urinary incontinence”, ‘‘ risk of impaired tissue integrity ‘‘ ‘‘impaired walking”, ‘difficulty in swallowing ‘‘ ‘‘deficit in toileting” ‘‘risk for ab-ingestis pneumonia ‘‘.

Higher risk for acute hospitalization was associated with younger age (OR 0.94, 95% CI 0.91–0.97), having a vascular access device (OR 2.33, 95% CI 1.02–5.36), impaired walking (OR 2.50, 95% CI 1.03–6.06). A lower risk for acute hospitalization was associated with a higher Conley score (OR 0.84, 95% CI 0.72–0.98) (Table 4).

**Table 4:** Logistic regression model to assess factors associated with acute hospitalization during CH stay.

	P	OR	Confidence Interval (95%)	
Age	0.001	0.94	0.91	0.97
Vascular access device	0.046	2.33	1.02	5.36
Conley score	0.017	0.84	0.72	0.98
Impaired walking	0.039	2.50	1.03	6.06

Model adjusted for: infective comorbidity, risk of infection”, ‘‘Constipation ‘‘; ‘Risk for impaired skin integrity”,” self-care deficit in toileting”, ‘‘risk for falling”.

## Discussion

The finding of this study identified an association between patient characteristics assessed using a standardized nursing assessment and outcomes of mortality and acute hospitalization during CH stay. Overall, patients admitted to a CH had a high mean age >80 years, high level of disability and greater nursing needs.

Increased risk of death was correlated with female sex, risk of pressure sores, risk of falling and having a vascular access device. Higher risk for acute hospitalization were associated with lower age, having a vascular access device, risk of falling and some nursing diagnoses such as self-care deficit in toileting and impaired walking. These results underlined the potential role of a standardized multidimensional nursing assessment to identify patients at high risk of adverse outcomes.

There is growing evidence about the role of nurses on patient outcomes in the hospital setting [19, 20], the residential care setting [21] and the primary health care setting [22]. However, scarce evidence has determined their role in transitional care setting, such as in CH, although it is a setting where nurses play a very important role.

CH is a transitional care solution to promote a rapid patient discharge from the hospital to a protected environment, to obtain clinical stabilization [1, 2]. Older adults are the main users of CH. They require long length of stay, have multiple needs and are at risk of adverse outcomes, such as pressure sores, infection, and nearness to end of life. The results of this study are consistent with this perspective. Equivalent or improved outcomes were described in community hospitals compared with acute hospital settings for mortality, readmissions and number of bed-days spent in hospital and functional independence at discharge. In CHs, nurses are the main providers of care due they hold managerial and patient-related responsibility than in acute hospitals [6].

A standardized multidimensional approach at admission allows us to identify the patients at risk of adverse outcomes. There is strong evidence that a multidimensional assessment is beneficial especially for older frail patients and its efficacy was largely demonstrated using a comprehensive geriatric assessment model [23].

Functional status, risk of falling and the presence of a vascular access device are variables associated with both mortality and acute hospitalization during CH stay, which could be a possible confounder.

Previous studies demonstrate a strong association between nursing assessments and mortality. Rothman et al. perform an observational study on a cohort of hospitalized patients over a 2-years period. They find that nursing assessments are strongly correlated with lower in-hospital and post-discharge mortality and conclude that nursing assessments can aid in physician care and possibly reduce hospital patient mortality, and they encourage more research in this field [24]. The present study adds further knowledge on this topic and confirms the importance of nursing assessment in evaluating the risk of patients’ adverse outcome development.

Older patients with a higher risk of pressure sores and several disabilities are less likely to be hospitalized. This could depend on the rate of subjects that were closer to the end of life. Previous studies in Nursing Home have shown that hospitalization at the end of life is often not beneficial [25]. Studies on end-of-life care suggested that the community hospital are perceived as preferable than acute hospital for patients and their relatives [5].

### Limitations

This was a retrospective single center study. However, data are from 2 years of observation, and the nursing assessment was performed according to international nursing guidelines.

The nursing assessment did not include any tool to assess frailty in our patients. This was a limitation, due to the well-established association between frailty and mortality [26, 27] and acute hospitalization [28, 29].

## Conclusion

Community Hospital cares for a mainly older population with multiple needs, one in five died during the CH stay. Using a multidimensional nursing assessment enabled the identification of mortality and acute hospitalization risk to target care and treatment. The present study adds further knowledge on this topic and confirms the importance of nursing assessment to evaluate the risk of patients’ adverse outcome development.

## Supporting information

S1 Checklist STROBE (strengthening the reporting of observational studies in epidemiology) checklist. (DOCX)

S1 Appendix Complete list of NANDA codes ordered by frequency. (DOCX)

S2 Appendix Complete list of NANDA codes among subjects died or hospitalized. (DOCX)
