# Supplementary material for: Predictors of adverse outcomes using a multidimensional nursing assessment in an Italian community hospital
Source: PLoS One. 2021 Apr 15;16(4):e0249630. doi: 10.1371/journal.pone.0249630 (PMC8049226; doi:10.1371/journal.pone.0249630)
Supplement: S1 Appendix — (DOCX) [file pone.0249630.s002.docx]

S1 Appendix. Complete list of NANDA codes ordered by frequency

| **Nursing diagnoses** | N(%) |
| --- | --- |
| Risk for infection | 204 (68.5) |
| Risk for falls | 196 (65.8) |
| Deficit in bathing self care | 186 (62.4) |
| Impaired walking | 179 (60.1) |
| Constipation | 161 (54.0) |
| Risk for impaired skin Integrity | 152 (51.0) |
| Impaired transfer ability | 149 (50.0) |
| Insomnia | 121 (40.9) |
| Risk for unstable blood glucose level | 109 (36.6) |
| Bowel incontinence | 77 (25.8) |
| Difficulty in swallowing | 70 (23.5) |
| Total urinary incontinence | 69 (23.1) |
| Impaired urinary elimination | 60 (20.1) |
| Self care deficit in dressing | 57 (19.1) |
| Self care deficit in toileting | 49 (16.4) |
| Risk for self-mutilation | 44 (14.7) |
| Impaired tissue integrity | 41 (13.7) |
| Deficient fluid volume | 40 (13.4) |
| Impaired gas exchange | 36 (12.1) |
| Ineffective breathing pattern | 31 (10.4) |
| Risk for disuse syndrome | 28 (9.4) |
| Ineffective self health management | 22 (7.4) |
| Acute pain | 21 (7.0) |
| Anxiety | 21 (7.0) |
| Impaired skin integrity | 17 (5.7) |
| Self care deficit in feeding | 15 (5.0) |
| Diarrhea | 12 (4.0) |
| Caregiver role strain | 12 (4.0) |
| Chronic pain | 12 (4.0) |
| Imbalaced nutrition less than body requirements | 10 (2.9) |
| Ineffective family therapeutic regimen management | 10 (2.9) |
| Unilateral neglect | 7 (2.3) |
| Risk for deficient fluid volume | 6 (2.0) |
| Urinary retention | 5 (1.7) |
| Nausea | 4 (1.3) |
| Acute confusion | 3 (1.0) |
| Effective drugs management | 2 (0.7) |
| Post traumatic syndrome (violence) | 2 (0.7) |
| Risk for constipation | 1 (0.3) |
| Impaired physical mobility | 1(0.3) |
| Risk for imbalanced body temperature | 1(0.3) |
| Hyperthermia | 1(0.3) |
| Powerlessnes | 1(0.3) |
| Death anxiety | 1(0.3) |
| Moral distress | 1(0.3) |
| Impaired oral mucose membrane | 1(0.3) |
| Decreased cardiac output | 1(0.3) |
| Ineffective airway clerance | 1(0.3) |
| Risk for Injury | 1(0.3) |
