# Supplementary material for: Predictors of adverse outcomes using a multidimensional nursing assessment in an Italian community hospital
Source: PLoS One. 2021 Apr 15;16(4):e0249630. doi: 10.1371/journal.pone.0249630 (PMC8049226; doi:10.1371/journal.pone.0249630)
Supplement: S2 Appendix — (DOCX) [file pone.0249630.s003.docx]

S2 Appendix. Complete list of nursing diagnoses among subjects died or acute hospitalized.

|  | **Mortality** | |  | **Acute hospitalization** | |  |
| --- | --- | --- | --- | --- | --- | --- |
|  | **Survivors**  **N=233** | **Not survivors**  **N=65** | **P** | **None Hospital admission**  **N=258** | **Acute hospitalization**  **N=40** | **P** |
| **Nursing diagnoses** |  |  |  |  |  |  |
| Risk for ab-ingestis pneumonia | 25 (10.7) | 21 (32.3) | <0.001 | 38 (14.7) | 7 (17.5) | 0.600 |
| Imbalanced nutrition less than body requirements | 7 (3.0) | 3 (4.6) | 0.460 | 9 (3.5) | 1 (2.5) | 0.999 |
| Risk for infection | 146 (62.7) | 56 (86.1) | <0.001 | 171 (66.3) | 31 (77.5) | 0.158 |
| Risk for imbalanced body temperature | 0 (0) | 1 (1.5) | 0.219 | 1 (0.4) | 0 (0) | 0.999 |
| Hyperthermia | 0 (0) | 1 (1.5) | 0.219 | 1 (0.4) | 0 (0) | 0.999 |
| Constipation | 124 (53.2) | 37 (56.9) | 0.596 | 150 (58.1) | 11 (27.5) | <0.001 |
| Diarrhea | 8 (3.4) | 4 (6.2) | 0.303 | 8 (3.1) | 4 (10.0) | 0.063 |
| Bowel incontinence | 57 (24.5) | 20 (30.8) | 0.313 | 68 (26.4) | 9 (22.5) | 0.595 |
| Risk for constipation | 1 (0.4) | 0 (0) | 0.999 | 1 (0.4) | 0 (0) | 0.999 |
| Impaired urinary elimination | 51 (21.9) | 9 (13.8) | 0.149 | 52 (20.2) | 8 (20.0) | 0.973 |
| Total urinary incontinence | 46 (19.7) | 23 (35.4) | 0.009 | 61 (23.6) | 8 (20.0) | 0.611 |
| Urinary retention | 5 (2.1) | 0 (0) | 0.589 | 5 (1.9) | 0 (0) | 0.999 |
| Deficient fluid volume | 31 (13.3) | 9 (13.8) | 0.920 | 32 (12.4) | 8 (20.0) | 0.193 |
| Risk for deficient fluid volume | 4 (1.7) | 2 (3.1) | 0.615 | 6 (2.3) | 0 (0) | 0.999 |
| Decreased cardiac output | 1 (0.4) | 0 (0) | 0.999 | 1 (0.4) | 0 (0) | 0.999 |
| Impaired gas exchange | 28 (12.0) | 8 (12.3) | 0.958 | 31 (12.0) | 5 (12.5) | 0.999 |
| Ineffective airway clearance | 1 (0.4) | 0 (0) | 0.999 | 0 (0) | 1 (2.5) | 0.135 |
| Ineffective breathing pattern | 23 (9.9) | 8 (12.3) | 0.577 | 30 (11.6) | 1 (2.5) | 0.095 |
| Risk for injury | 1 (0.4) | 0 (0) | 0.999 | 1 (0.4) | 0 (0) | 0.999 |
| Risk for disuse syndrome | 23 (9.9) | 5 (7.7) | 0.588 | 27 (10.5) | 1 (2.5) | 0.146 |
| Impaired tissue integrity | 37 (15.9) | 4 (6.1) | 0.043 | 30 (11.6) | 11 (27.5) | 0.007 |
| Impaired oral mucous membrane | 1 (0.4) | 0 (0) | 0.999 | 1 (0.4) | 0 (0) | 0.999 |
| Impaired skin integrity | 14 (6.0) | 3 (4.6) | 0.999 | 14 (5.4) | 3 (7.5) | 0.711 |
| Risk for impaired skin Integrity | 110 (47.2) | 42 (64.6) | 0.013 | 136 (52.7) | 16 (40.0) | 0.134 |
| Caregiver Role Strain | 6 (2.6) | 4 (6.1) | 0.303 | 9 (3.5) | 3 (7.5) | 0.210 |
| Risk for Caregiver Role Strain | 1 (0.4) | 0 (0) | 0.999 | 0 (0) | 1(2.5) | 0.135 |
| Ineffective self- health management | 17 (7.3) | 5(7.7) | 0.999 | 21 (8.1) | 1 (2.5) | 0.330 |
| Ineffective family therapeutic regimen management | 6 (2.6) | 4 (6.1) | 0.234 | 8 (3.1) | 2 (5.0) | 0.629 |
| Effective drugs management | 1(0.4) | 11 (1.5) | 0.390 | 2 (0.8) | 0 (0) | 0.999 |
| Impaired physical mobility | 0 (0) | 11 (1.5) | 0.219 | 1 (0.4) | 0 (0) | 0.999 |
| Impaired Walking | 144 (61.8) | 35 (53.8) | 0.247 | 150 (58.1) | 29 (72.5) | 0.084 |
| Impaired transfer ability | 116 (49.8) | 32 (49.2) | 0.999 | 128 (49.6) | 20 (50.0) | 0.999 |
| Insomnia | 94 (40.3) | 27(41.5) | 0.882 | 106 (41.1) | 15 (37.5) | 0.654 |
| Self care deficit feeding | 118 (50.6) | 33 (50.7) | 0.989 | 134 (51.9) | 17 (42.5) | 0.257 |
| Difficulty in swallowing | 45 (19.3) | 27 (41.5) | <0.001 | 63 (24.4) | 9 (22.5) | 0.729 |
| Bathing self care Deficit | 149 (63.9) | 37 (56.9) | 0.282 | 160 (62.0) | 26 (65.0) | 0.739 |
| Dressing self-care Deficit | 49 (21.0) | 8 (12.3) | 0.111 | 47 (18.2) | 10 (25.0) | 0.316 |
| Self-care deficit toileting | 43 (18.4) | 6 (9.2) | 0.074 | 35 (13.7) | 14 (35.0) | 0.001 |
| Unilateral Neglect | 7 (3.0) | 0 (0) | 0.156 | 6 (2.3) | 1 (2.5) | 0.999 |
| Powerlessness | 1 (0.4) | 0 (0) | 0.999 | 1 (0.4) | 0 (0) | 0.999 |
| Acute Confusion | 3 (1.3) | 0 (0) | 0.999 | 1 (0.4) | 2 (5.0) | 0.049 |
| Acute Pain | 18 (7.7) | 3 (4.6) | 0.584 | 18 (6.9) | 3 (7.5) | 0.999 |
| Chronic Pain | 12 (5.1) | 0 (0) | 0.075 | 9(3.5) | 3 (7.5) | 0.210 |
| Nausea | 2 (0.8) | 2 (3.1) | 0.209 | 4 (1.6) | 0 (0) | 0.999 |
| Risk for self-mutilation | 23 (9.9) | 21 (32.3) | <0.001 | 37 (14.3) | 7 (17.5) | 0.607 |
| Post traumatic sindrome (violence) | 2 (0.8) | 0 (0) | 0.999 | 2 (0.8) | 0 (0) | 0.999 |
| Anxiety | 19 (8.1) | 2 (3.1) | 0.270 | 19 (7.4) | 2 (5) | 0.750 |
| Death anxiety | 1 (0.4) | 0 (0) | 0.999 | 0 (0) | 1 (2.5) | 0.135 |
| Risk for falls | 155 (66.5) | 42 (64.6) | 0.774 | 175 (67.8) | 22 (55.0) | 0.111 |
| Moral distress | 1 (0.4) | 0 (0) | 0.999 | 1 (0.4) | 0 (0) | 0.999 |
| Risk for unstable blood glucose level | 83 (35.6) | 26 (40.0) | 0.562 | 95 (36.8) | 14 (35.0) | 0.810 |
